# Sparse Self-Prompt-Guided Stereo Matching for Real-World Generalization

**DOI:** 10.3390/s26103173

**Published:** 2026-05-17

**Authors:** Hangbiao Li, Haojun Mo, Xing Li, Tao Fang, Sikun Liu, Shuzhen Yu, Zhibo Rao

**Affiliations:** 1 School of Information and Engineering, Nanchang Hangkong University, Nanchang 330063, China; 13117802085@163.com (H.L.); lixing36@foxmail.com (X.L.); shuzhenyu@buaa.edu.cn (S.Y.); 2 North Lian Chuang Communication Co., Ltd., Nanchang 330096, China; mo15270021365@163.com (H.M.); ftnupt@foxmail.com (T.F.); 3 School of Computer Science and Technology, University of Science and Technology of China, Hefei 230026, China; liusikun@mail.ustc.edu.cn; 4 School of Instrumentation and Optoelectronic Engineering, Beihang University, Beijing 100191, China

**Keywords:** stereo matching, domain generalization, vision foundation models, sparse prompt, real-world perception, disparity estimation

## Abstract

Stereo matching has witnessed rapid advances on curated benchmarks, yet deploying models in unconstrained real-world environments remains a fundamental challenge. This paper presents a sparse self-prompt-guided network (SSPGNet) for stereo matching with strong generalization across diverse environments. Our core innovation lies in a sparse self-prompt guidance mechanism: (1) a sparse disparity map, used as a prompt, is self-estimated from visual foundation model features via cost aggregation; (2) the sparse disparity is progressively refined into dense disparity maps through cross-attention-based stereo feature interaction, enabling sparse-to-dense disparity prediction. Additionally, we collected a diverse set of indoor and outdoor stereo pairs by using a ZED 2 camera to assess the real-world performance of our model. Extensive experiments demonstrate that the proposed sparse-to-dense prompt mechanism not only preserves the semantic awareness of visual foundation models but also enhances stereo correspondence reasoning, achieving strong performance on public benchmarks and our in-the-wild dataset. Specifically, under the cross-domain (zero-shot) protocol, the proposed SSPGNet achieves bad-pixel error rates of 3.6% on KITTI 2012 (>3 px), 4.4% on KITTI 2015 (>3 px), 7.6% on Middlebury (>2 px), and 2.1% on ETH3D (>1 px), ranking first on three of the four public benchmarks. These results highlight the potential of SSPGNet for direct deployment in real-world stereo perception systems. The code is publicly available at GitHub.

## 1. Introduction

Stereo matching has been studied for nearly half a century and remains a fundamental challenge in computer vision and robotics. Deep learning and large-scale datasets have driven significant progress, resulting in the proliferation of deep stereo matching models [1,2]. Although these models achieve state-of-the-art performance [3,4] on many public benchmarks—even without fine-tuning [5]—they are still rarely adopted in real-world applications. In other words, current methods excel at predicting disparity maps on specific benchmark datasets but still struggle in open-world scenarios. In contrast, foundation models have emerged in related areas and have demonstrated strong performance on in-the-wild images, such as DepthAnything [6] and Segment Anything [7]. Meanwhile, recent engineering-oriented studies in Sensors further highlight the practical value of stereo vision and disparity estimation in real-world ranging and deployment scenarios [8]. This raises a critical question: how can stereo matching achieve a level of real-world generalization comparable to that of DepthAnything [6]?

DepthAnything [6] suggests that scaling is a key factor in achieving zero-shot generalization, and its success benefits substantially from vision foundation models (VFMs). The performance improvement comes primarily from robust visual features extracted by VFMs such as DINO v2 [9], SAM [7], and DepthAnything [6]. Intuitively, stereo matching should benefit from such robust visual features if they can effectively capture pixel-level spatial information or provide a unified representation across two views. If this assumption holds, we could compare left–right features by constructing a cost volume and then regress the disparity map. However, in practice, we do not obtain the expected results because VFM tokens are spatially coarse and cannot directly preserve sufficient coordinate information for stereo correspondence.

This paper introduces a sparse self-prompt-guided network (SSPGNet) to address the challenges of stereo matching in dynamic, open-world environments. Our key innovation lies in incorporating geometric priors into the stereo matching network through a sparse self-prompt, thereby enhancing its robustness. Specifically, our approach begins by extracting robust features from VFMs and exploring the relationship between tokens and pixel coordinates. We then estimate a sparse disparity map from the left–right features. Using this sparse disparity as an initial prompt, we guide the model to focus its learning capacity on the most informative regions of the stereo pair. The second key component is cross-attention-based stereo feature interaction. We employ cross-shift window attention between stereo features to generate an affinity matrix, which progressively refines the sparse disparity into a dense map through spatial propagation. This design provides a more nuanced understanding of disparity relationships, enabling the model to handle complex, occluded, and real-world environments more effectively.

**Figure 1:**
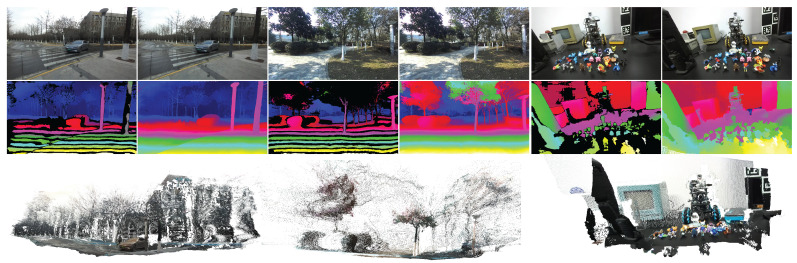
Example predictions on in-the-wild stereo pairs. We show stereo pairs, sparse self-prompts, disparity maps, and reconstructed 3D models, demonstrating that our model predicts high-quality disparities and generalizes well to in-the-wild stereo pairs. More results on in-the-wild scenes and public datasets are provided in the Appendix A.

To validate the robustness and stability of SSPGNet, we evaluate our model on more than eight public datasets, including CreStereo [10], SceneFlow [11], KITTI 2012 and 2015 [12,13], ETH3D [14], Middlebury [15], and Flickr1024 [16]. Moreover, we collected a dataset of stereo pairs from diverse indoor and outdoor environments using a ZED 2, as shown in Figure 1. Through extensive experiments, we demonstrate that SSPGNet not only outperforms state-of-the-art methods on standard benchmarks but also achieves strong generalization on our in-the-wild dataset, highlighting its practical value. In summary, our work makes the following contributions:

- We design a sparse self-prompt mechanism based on vision foundation models to achieve strong generalization for in-the-wild stereo matching.
- We investigate whether tokens from vision foundation models can represent the coordinate information of all pixels within the original patch for stereo matching.
- We collect a set of in-the-wild stereo pairs and evaluate our model on more than eight public datasets, highlighting its potential for direct deployment in real-world stereo perception systems.

## 2. Related Work

Deep Stereo Matching. Since GC-Net [17], many end-to-end models have followed a common paradigm that includes feature extraction, cost volume construction, cost aggregation, and disparity regression. Shen et al. constructed a cascade pyramid cost volume and regressed a high-quality disparity map from coarse to fine [18]. Li et al. designed a hierarchical network with recurrent refinement to update disparities from coarse to fine and proposed a group correlation layer to address erroneous rectification [10]. Zhao et al. designed a decoupled long short-term memory (LSTM) module to separate the hidden state from the update matrix of disparity maps [19]. Guan et al. proposed a disparity proposal network to adaptively prune the disparity search space [20]. Wang et al. aggregated hidden disparity across multiple frequencies, mitigating the risk of losing important hidden disparity during iterative processes [21]. Zhou et al. proposed a consistency-aware self-training framework to leverage real-world unlabeled data in a teacher–student manner [22]. Wang et al. proposed an adaptive down-sampling module and a disparity alignment module to balance accuracy and efficiency, enabling real-time stereo matching with reduced computational cost [23].

Domain-Generalized Stereo Matching. Zhang et al. revisited feature consistency using a contrastive feature loss function and achieved superior performance on four datasets [24]. Rao et al. introduced masked modeling into stereo matching to build a pseudo-multi-task learning framework for more stable cross-domain performance [25]. Zhang et al. introduced vision foundation models into the stereo matching pipeline and proposed a cosine-constrained concatenation cost to improve zero-shot performance [26]. Wen et al. constructed a large-scale synthetic training dataset with high diversity and strong photorealism to achieve strong zero-shot generalization [27]. Bartolomei et al. seamlessly integrated stereo matching with learned contextual cues to effectively handle critical challenges [28]. Jiang et al. incorporated a robust monocular relative depth model into a recurrent stereo-matching framework to recover accurate disparity [29]. Cheng et al. used confidence-based guidance to adaptively select reliable stereo cues for mono-depth scale-shift recovery and then guide stereo estimation in ill-posed regions [30]. Zhang et al. studied the synthetic-to-real fine-tuning robustness of stereo and optical-flow networks and proposed a knowledge-transfer framework that preserves cross-domain generalization while adapting the network to a target real-world domain [31].

Sparse Prompt Models. Jia et al. introduced visual prompt tuning as an alternative to full fine-tuning for large-scale transformer models in vision tasks [32]. Kirillov et al. proposed the Segment Anything Model (SAM), which can be prompted by points and other visual prompts to achieve strong generalization ability [7]. Shen et al. generated sparse yet reliable pseudo-labels to reduce the domain gap [33]. Yang et al. proposed sparse visual domain prompts to optimize prompt parameters differently for each sample, facilitating efficient adaptation to the target domain [34]. Guo et al. presented flexible structure control with temporally sparse signals, requiring only one or a few inputs [35]. Li et al. proposed a promptable interactive segmentation model based on sparse prompts, such as points, boxes, and scribbles [36]. Huang et al. proposed a sparse-to-dense localization pipeline by leveraging powerful monocular foundation models to provide dense supervision for depth completion [37].

Vision Foundation Models. He et al. developed an asymmetric encoder–decoder architecture with masked representation learning via supervised pre-training [38]. Liu et al. proposed a grafted feature method that uses cosine similarity as a bridge to address domain shift [39]. Zhang et al. leveraged the zero-shot capacity of a foundation model to alleviate the cross-domain generalization problem in stereo matching [26]. Yang et al. exploited large-scale unlabeled data to achieve impressive generalization ability for depth prediction [6]. Yang et al. further replaced all labeled real images with synthetic images and scaled up the capacity of the teacher model, achieving better metric depth models [40]. Liu et al. utilized implicit prior knowledge to better adapt to task characteristics and unleash the potential of the SAM [41]. Liang et al. proposed a two-stage knowledge distillation framework that leverages powerful monocular foundation models to provide dense supervision for depth completion [42]. Wang et al. integrated a vision foundation model with feature-pyramid representation and a data-augmentation mechanism into a self-supervised stereo framework, achieving strong robustness in both self-supervised and supervised regimes [43].

We summarize the representative methods discussed above and the gap each one leaves with respect to SSPGNet in Table 1.

## 3. Methodology

### 3.1. Overview

**Figure 2:**
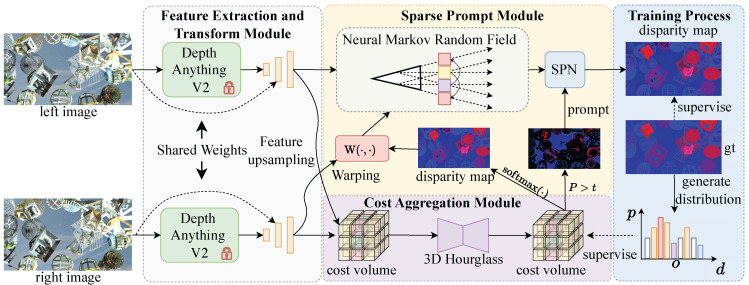
Pipeline of the proposed method. We first use stereo features extracted by the foundation model to generate a sparse disparity prompt. Then, we use this prompt to guide the sparse prompt module, achieving sparse-to-dense disparity prediction.

Given a left–right stereo pair $I_{L,R}\in R^{3\times H\times W}$, the goal of stereo matching is to estimate the correspondence information, namely, the disparity map, between the stereo images, where H×W denotes the image size. In this paper, we aim to estimate disparity maps for stereo pairs captured in the wild. As shown in Figure 2, our model contains five core parts: a feature extraction module, a feature transform module, a cost volume construction module, a cost aggregation module, and a sparse prompt module.

**Figure 3:**
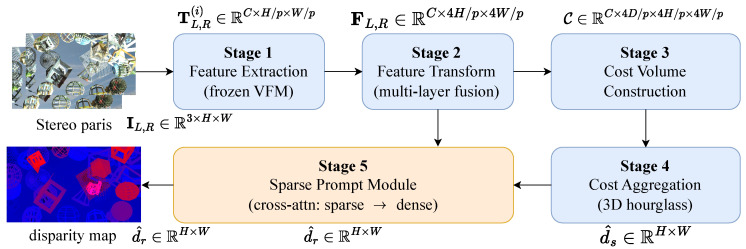
Stage-by-stageworkflow of the proposed method. We decompose the pipeline into five sequential stages: **(S1)** feature extraction with a frozen vision foundation model, **(S2)** multi-layer feature transform that fuses VFM tokens into transformed features, **(S3)** cost volume construction from the left/right features, **(S4)** 3D hourglass cost aggregation that produces a sparse/blurred disparity, and **(S5)** the proposed sparse prompt module that turns the sparse disparity into the final dense disparity via cross-attention. The tensor shown above each inter-stage edge denotes the output of the preceding stage (e.g., $T_{L,R}^{\left(i\right)}$ is the output of S1, $F_{L,R}$ is the output of S2, ${\hat{d}}_{r}$ is the final output of S5). The purple skip-edge indicates that S5 also takes $F_{L,R}$ from S2 as a cross-attention input, in addition to ${\hat{d}}_{s,b}$ from S4.

As shown in Figure 3, the data flow proceeds in five stages. First, we use a vision foundation model to extract token features $T_{L,R}\in R^{C\times H/p\times W/p}$ with patch size p×p from stereo pairs while keeping the parameters of the vision foundation model fixed throughout training. Next, we employ the feature transform module to reassemble features from different stages of the foundation model into large-scale features $F_{L,R}\in R^{C\times 4H/p\times 4W/p}$. Then, we construct a cost volume from the transformed features and feed it into a cost aggregation module based on a 3D hourglass network, generating high-confidence sparse or blurred disparity maps ${\hat{d}}_{s,b}\in R^{H\times W}$. Finally, through cross-attention-based stereo feature interaction in the sparse prompt module, we progressively refine sparse disparity into dense disparity maps ${\hat{d}}_{r}\in R^{H\times W}$, achieving sparse-to-dense disparity prediction. We introduce each part in the following sections.

### 3.2. Model Architecture

**Feature Extraction Module.** Vision foundation models have demonstrated strong performance on multiple tasks, especially on depth prediction. Therefore, we use the empirical configurations of vision foundation models (DINO v2 [9] or DepthAnything v2 [40]) as our feature extraction module. DepthAnything v2 uses DINO v2 based on ViT [44] as its encoder, so DepthAnything v2 and DINO v2 share the same backbone structure. Following DepthAnything v2, we extract tokens from stereo pairs $I_{L,R}$ at different layers from shallow to deep: $T_{L,R}^{\left(i\right)}\in R^{C\times H/p\times W/p},i\in \left\{4,\;11,\;17,\;23\right\}$, where the patch size *p* is set to 14. These four indices are roughly equally spaced across the 24-layer ViT-L/14 backbone of DINOv2/DepthAnything v2 and follow the empirical configuration recommended by DepthAnything v2. Meanwhile, to avoid catastrophic forgetting, we freeze the parameters of the vision foundation models and train only the remaining modules. Then, using the feature transform module, we convert the small-sized tokens into larger-scale features.

**Figure 4:**
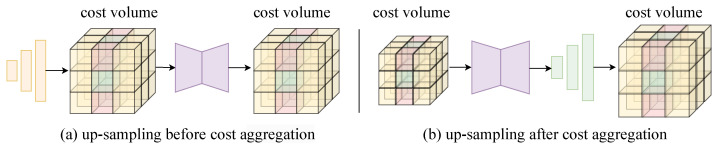
Two ways to use tokens from foundation models. One way is to up-sample the tokens before cost aggregation. The other is to up-sample the cost volume after cost aggregation.

**Feature Transform Module.** Compared with conventional stereo features, the token resolution is too small, with only $\frac{H}{14}\times \frac{W}{14}$. Therefore, we up-sample and fuse tokens from different layers to obtain features $F_{L,R}\in R^{C\times 4H/p\times 4W/p}$. The process can be written as

(1) $$\begin{array}{c} F_{L,R}^{\left(i\right)}=\mathrm{FeatUp}\left(\mathrm{Conv}\left(T_{L,R}^{\left(i\right)}\right),I_{L,R}\right), \end{array}$$

where Conv(·) denotes a convolution with kernel size 1×1 used to adjust the channel number and FeatUp(·) denotes the feature up-sampling function guided by left–right images $I_{L,R}$. We then fuse these features to obtain the transformed features $F_{L,R}$ as follows:

(2) $$\begin{array}{c} F_{L,R}=\mathrm{Conv}\left(\left[F_{L,R}^{\left(i\right)},\ldots \right]\right),i\in \left\{4,\;11,\;17,\;23\right\}, \end{array}$$

where Conv(·) denotes a convolution with kernel size 1×1 used to fuse the features, and [·,·] denotes concatenation along the channel dimension. A natural question arises here: can a token represent the coordinate information of all pixels within the original patch, and if so, why is token up-sampling still necessary? Unlike other dense prediction tasks such as semantic segmentation or depth prediction, stereo matching is fundamentally a correspondence problem. Therefore, pixel-level coordinate information is crucial. To investigate this issue, we design a verification scheme. As shown in Figure 4, an alternative is to decode a smaller cost volume directly from the tokens $T_{L,R}$ using a series of 3D deconvolutions. Therefore, in the experiments, we compare constructing the cost volume from transformed features with constructing it directly from tokens.

**Cost Volume Construction.** Given the transformed features $F_{L,R}\in R^{C\times 4H/p\times 4W/p}$, we construct the cost volume $C\left(d,h,w\right)\in R^{C\times 4D/p\times 4H/p\times 4W/p}$ by concatenation as follows:

(3) $$\begin{array}{c} C\left(d,h,w\right)=[F_{L}\left(h,w\right),F_{R}\left(h,w-d\right)], \end{array}$$

where [·,·] denotes concatenation along the channel dimension, *D* denotes the maximum disparity range, and d∈{1, 2, …, 4D/p} is the disparity index.

**Cost Aggregation Module.** We use the cost aggregation module to obtain the initial disparity maps and probability distributions, thereby producing a high-quality sparse prompt. Following previous works, we adopt a 3D hourglass network [45] as the cost aggregation module. The cost aggregation process can be formulated as

(4) $$\begin{array}{c} {\hat{C}}_{i}=\mathrm{Hourglass}\left(C_{i}\right),i\in \left\{1,\;2,\;3\right\}, \end{array}$$

(5) $$\begin{array}{c} {\hat{P}}_{i}=\mathrm{Softmax}\left({\hat{C}}_{i}\right),i\in \left\{1,\;2,\;3\right\}, \end{array}$$

(6) $$\begin{array}{c} {\hat{d}}_{i}=\sum_{d=1}^{4D/p}d\cdot {\hat{P}}_{i}\left(d\right),\;i\in \left\{1,\;2,\;3\right\}, \end{array}$$

where ${\hat{C}}_{i}\in R^{D\times H\times W}$ denotes the *i*-th cost volume after the 3D hourglass, ${\hat{P}}_{i}$ denotes the probability distribution of the *i*-th cost volume, and ${\hat{d}}_{i}$ represents the *i*-th disparity map.

Next, we use the probability distribution of the final stage to generate the sparse prompt. In real-world scenes, the probability distribution is often multi-modal [46], and previous work has proposed an effective distribution modeling method for this case. The multi-modal probability distribution $P_{gt}\in R^{D\times H\times W}$ can be modeled as a mixture of Laplacians based on the ground-truth disparity $d_{gt}$, as follows:

(7) $$\begin{array}{c} P_{gt}\left(d_{gt}\right)=\sum_{k=1}^{K}w_{k}\cdot {\mathrm{Laplacian}}_{\mu_{k},b_{k}}\left(d_{gt}\right), \end{array}$$

where *K* denotes the number of disjoint subsets, and $w_{k}$, $\mu_{k}$, and $b_{k}$ are the weight, mean, and scale parameters of the *k*-th Laplacian distribution, respectively. We use the default setting in [46] to supervise the predicted probability distributions. Then, we apply a confidence threshold t∈{0.08, 0.10, 0.12, 0.15} to the probability distribution ${\hat{P}}_{3}$ of the final 3D hourglass output to obtain sparse disparity maps ${\hat{d}}_{s}$ as prompts. The filtering process is defined as

(8) $$\begin{array}{c} {\hat{d}}_{s}=f\left({\hat{P}}_{3},t\right)\cdot {\hat{d}}_{3}, \end{array}$$

where f(·) denotes the indicator function.

**Sparse Prompt Module.** Recent works have emphasized that vision foundation models can extract structural information from the input. Meanwhile, adjacent pixels usually have similar disparity values. Therefore, we use such structural information to achieve sparse-to-dense propagation. First, we extract a latent feature space z from the left-transformed feature $F_{L}\left(i,j\right)$ and the warped right-transformed feature $F_{R}\left(i-\hat{d},j\right)$ through a Neural Markov Random Field (NMRF) based on cross-shift window attention, where i,j denote pixel coordinates. Then, we convert the latent feature space z into an affinity matrix $A\in R^{8\times H\times W}$ using a one-layer multi-layer perceptron (MLP). Finally, we embed the sparse disparity map ${\hat{d}}_{s}$ into a hidden representation $H\in R^{8\times H\times W}$ and update this hidden representation using the affinity matrix A, thereby obtaining the sparse-to-dense disparity map ${\hat{d}}_{r}$ through a spatial propagation network (SPN). The process can be written as:

(9) $$\begin{array}{c} z=\mathrm{NMRF}(F_{L}\left(i,j\right),F_{R}\left(i-\hat{d},j\right)), \end{array}$$

(10) $$\begin{array}{c} A=\mathrm{MLP}(z), \end{array}$$

(11) $$\begin{array}{c} H=\mathrm{Embed}({\hat{d}}_{s}), \end{array}$$

(12) $$\begin{array}{c} {\hat{d}}_{r}=\mathrm{SPN}\left(H,A\right). \end{array}$$

Therefore, we obtain the dense disparity map after the sparse prompt module.

### 3.3. Loss Functions

We use the smooth $L_{1}$ loss $L_{s}$ to supervise the predicted disparity maps, as follows:

(13) $$\begin{array}{c} L_{s}=\sum_{i=1}^{I=4}{\mathrm{smooth}}_{L_{1}}\left(d_{gt}-{\hat{d}}_{i}\right), \end{array}$$

where $d_{gt}$ denotes the ground-truth disparity map, ${\hat{d}}_{i}$ represents the *i*-th predicted disparity, and *I* denotes the total number of predicted maps. Meanwhile, we employ a cross-entropy loss $L_{ce}$ to supervise the probability distributions of the cost volume, as follows:

(14) $$\begin{array}{c} L_{ce}=\sum_{i=1}^{I=3}\left(-\frac{1}{N}\sum_{d}P_{gt}\left(d\right)\log{\hat{P}}_{i}\left(d\right)\right), \end{array}$$

Note that the cross-entropy term is summed from i=1 to 3 rather than 4 because only the three cost-aggregation stages produce explicit probability distributions over disparity (${\hat{P}}_{1,2,3}$). The fourth disparity output ${\hat{d}}_{r}$ is obtained directly via spatial propagation in the sparse prompt module and therefore has no associated distribution to supervise. ${\hat{P}}_{i}$ denotes the *i*-th predicted distribution, $P_{gt}$ is the ground-truth distribution obtained from the normalized multi-modal Laplacian distribution of $d_{gt}$, and *N* denotes the number of pixels in the image. Thus, the total loss $L_{total}$ is defined as follows:

(15) $$\begin{array}{c} L_{total}=L_{s}+L_{ce}. \end{array}$$

## 4. Experiments

We conduct a comprehensive set of experiments to validate the proposed framework. Our evaluation is designed to answer three key questions: (1) How does our method perform against state-of-the-art techniques across standard benchmarks? (2) How do feature size and the quality of the generated sparse labels affect performance? (3) How does our method perform in the wild with a ZED 2 camera?

### 4.1. Datasets and Evaluation Metrics

**Datasets.** We briefly describe these datasets from different domains. (1) Source datasets. We train all models only on the SceneFlow dataset [11] or the CreStereo dataset [10]. (2) Target domain. Following previous works [18,39], we evaluate all models without fine-tuning on KITTI 2012 and 2015 (KT-12 and KT-15) [12,13], ETH3D (ET) [14], and Middlebury (MB) [15]. Meanwhile, we collect stereo pairs using a ZED 2 to provide real-world stereo images for testing performance in the wild.

**Evaluation Metrics.** We evaluate our model using two standard metrics. (1) End-point error (EPE), which computes the average absolute error between the predicted disparity map and the ground truth. (2) The τ-pixel error, which measures the percentage of pixels with an absolute error greater than τ pixels. The official evaluation servers of KITTI 2012/2015 (https://www.cvlibs.net/datasets/kitti/eval_stereo.php, accessed on 13 May 2026), Middlebury (https://vision.middlebury.edu/stereo/eval3/, accessed on 13 May 2026), and ETH3D (https://www.eth3d.net/low_res_two_view, accessed on 13 May 2026) further report the disparity error in different regions, including all pixels, non-occluded pixels, and occluded pixels, denoted by *All*, *Noc*, and *Occ*, respectively.

### 4.2. Main Properties

**Figure 5:**
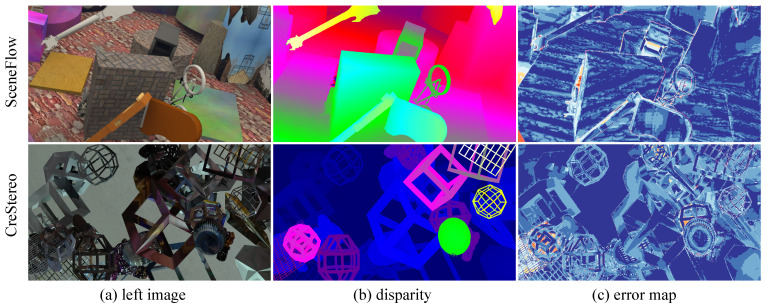
Example results in the source domain. Our model predicts clear disparity maps on the pre-training datasets.

**The Proposed Structure.** We compare the core parts of our model, especially the feature transform module (FTM) and sparse prompt module (SPM). As shown in Table 2 and Figure 5, three observations can be made: (1) compared with decoding a small-size cost volume, feature transformation is important for models based on vision foundation; (2) consistent with previous work, multi-scale aggregation helps the model perform better; and (3) the sparse prompt module further improves matching accuracy, demonstrating the effectiveness of our architecture. In addition, we infer that the key factor is that feature transformation reconstructs scale information, which is crucial to recovering location information. Large models convert patches into tokens, thereby reducing feature resolution and weakening positional cues. From this perspective, although large models provide strong semantic features, these features do not seem to contain sufficient positional information for stereo matching. Therefore, reconstructing coordinate information is essential when using large models for position-sensitive tasks.

**Figure 6:**
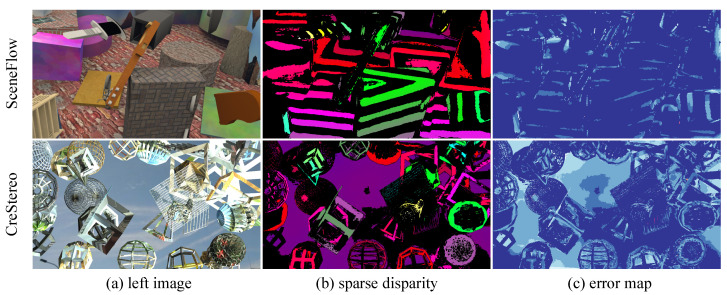
Sparse disparity in the source domain. It shows that the model can generate a high-quality prompt in the source domain.

**Figure 7:**
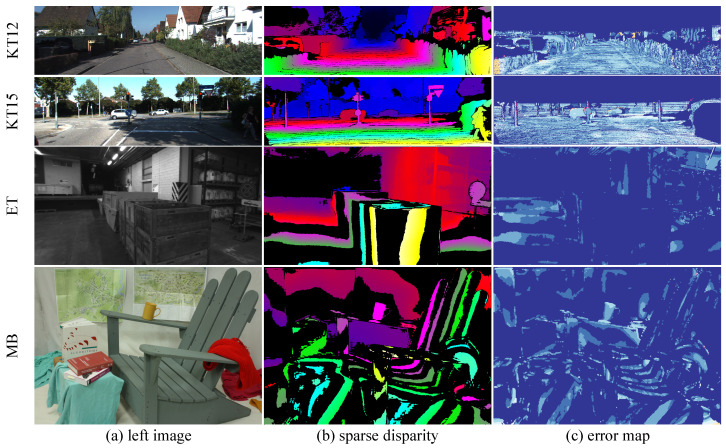
Sparse disparity in the cross-domain setting. It shows that the model can generate a high-quality prompt in the cross-domain setting.

**Sparse Disparity Map Evaluation.** We evaluate the sparse disparity maps produced by our model in both in-domain and cross-domain settings, as shown in Figure 6 and Figure 7. We also report quantitative results for sparse disparity in Table 3. Three conclusions can be drawn: (1) the sparse disparity maps are of high quality, with a 3-pixel error lower than 1.50% in the best setting; (2) compared with other uncertainty-based methods, the sparse disparity maps generated by our model are denser and more accurate; and (3) when comparing in-domain and cross-domain sparse disparity maps, the sparsity and accuracy do not degrade significantly, indicating that our model is robust. Overall, benefiting from vision foundation models, our method can generate stable sparse disparity maps to guide the sparse-to-dense process.

**Different Backbone.** We compare models trained with two vision foundation models, DINO v2 and DepthAnything v2, on two synthetic datasets (CreStereo and SceneFlow). As shown in Table 4, three conclusions can be drawn: (1) both vision foundation models help improve performance in in-domain and cross-domain settings; (2) compared with DINO v2, the performance gain from using DepthAnything v2 is more significant; and (3) the larger model achieves better generalization. In addition, we speculate that depth prediction and stereo matching are more closely related tasks, which is why DepthAnything v2 helps the model perform better in 3D perception. Therefore, our final model uses DepthAnything v2 as the feature extractor.

### 4.3. Results and Comparisons

**Figure 8:**
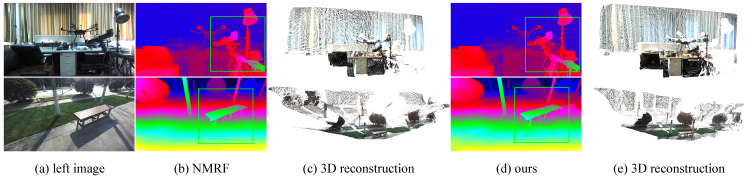
Example results in the wild. We collected stereo pairs in the wild using a ZED 2 camera to test the model’s generalization. The results show that our model can obtain good disparity predictions in the wild, indicating its practical applicability.

**Figure 9:**
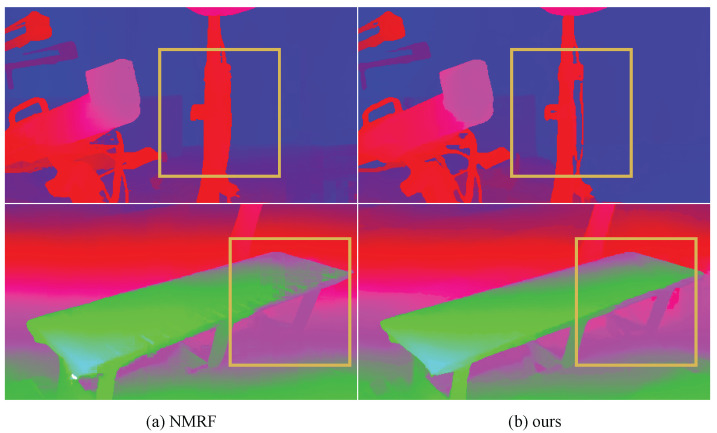
Zoomed-in view in the wild. The zoomed-in view of results shows that our method can predict disparity maps with finer details.

**In-the-Wild Generalization.** We employ a ZED 2 camera to capture binocular images in both indoor and outdoor environments to evaluate the model’s performance in real-world scenarios, as illustrated in Figure 8. To make the per-method differences in Figure 8 easier to perceive, we provide zoomed-in views of two representative scenes in Figure 9, in which boxed regions show that our model preserves thin structures (top row) and object boundaries (bottom row) more faithfully than the recent NMRF baseline [20]. The results demonstrate that our model can generate clear and accurate disparity maps across diverse environmental conditions. Notably, it maintains robust performance even in challenging scenarios, such as occlusions and varying lighting conditions. Thus, our model exhibits strong generalization capability and effectively adapts to different backgrounds and scene complexities without requiring additional fine-tuning. This suggests strong potential for deployment in practical applications such as autonomous navigation, robotics, and augmented reality, which is consistent with the application trend of recent sensor studies on deployable stereo systems [8]. More results in open environments are provided in the Appendix A. Overall, our model can reliably predict high-quality disparity maps in diverse settings.

**Figure 10:**
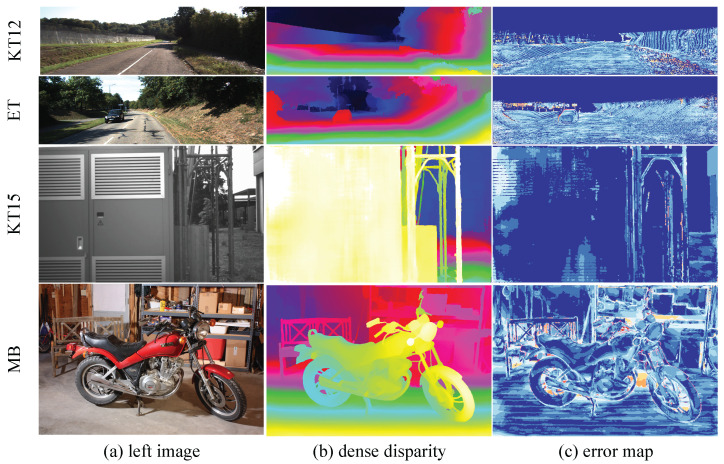
Predicted disparity in the cross-domain setting. It shows that our model predicts a dense disparity map in the cross-domain setting.

**Figure 11:**
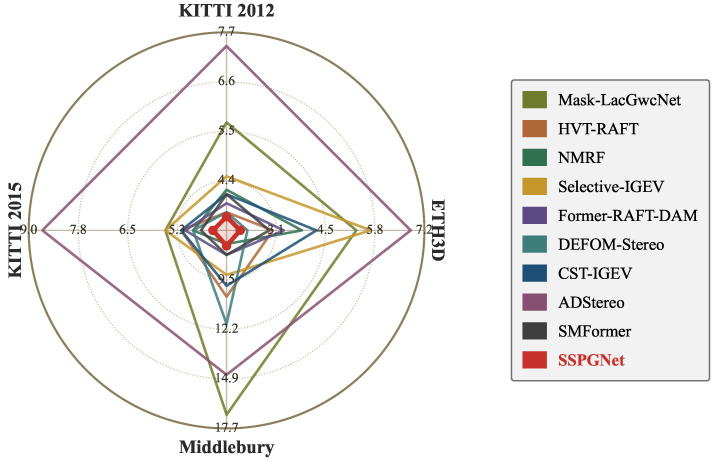
Radar visualization of peak cross-domain results (Table 5). The four spokes show the bad-pixel rate of each method on KITTI 2012, ETH3D, Middlebury, and KITTI 2015; the center of every spoke is the best observed value across the ten compared methods (lower error = closer to center). SSPGNet’s polygon (red) is the innermost on three of the four axes and ties with NMRF on Middlebury, summarizing its cross-domain advantage among 2023–2026 peer methods at a glance. All error values are bad-pixel rates in %.

**Cross-Domain Comparison.** We compare our model with state-of-the-art domain-generalized stereo matching methods and report quantitative cross-domain results on four public real-world datasets. Table 5 shows that our model achieves competitive peak cross-domain performance across the four datasets. Our model achieves the best performance on ETH3D and KITTI 2012 and 2015, and the second-best performance on Middlebury. Meanwhile, Figure 10 shows, for several cross-domain test pairs, the left image, our predicted dense disparity, and the corresponding error map, illustrating that SSPGNet produces dense disparity with low error in the cross-domain setting.The same comparison is also visualized in Figure 11: the four spokes plot the per-benchmark bad-pixel rate of ten 2023–2026 methods, and SSPGNet’s polygon (red) is the innermost on three of the four axes, summarizing the cross-domain advantage at a glance. Therefore, our model demonstrates strong generalization performance.

**Figure 12:**
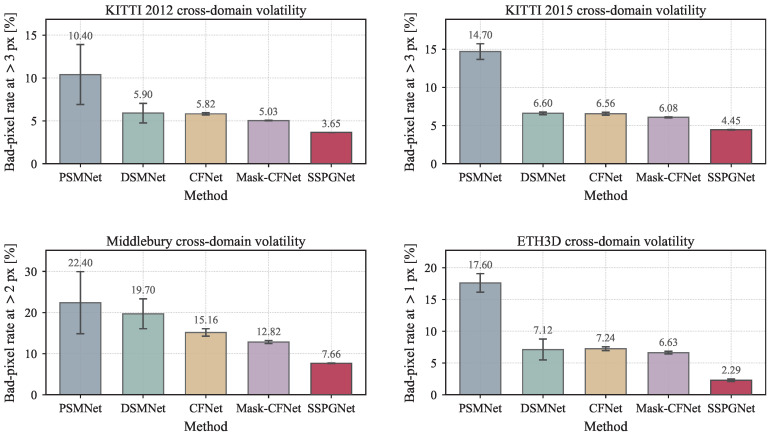
Visualization of cross-domain volatility (Table 6). Each panel shows the mean cross-domain bad-pixel rate (bar height) and one standard deviation across training epochs (whisker) for five representative methods on one of the four target benchmarks (KITTI 2012, KITTI 2015, Middlebury, and ETH3D). SSPGNet (right-most red bar in every panel) attains both the lowest mean and an almost-invisible standard deviation, indicating that its cross-domain generalization is achieved without sacrificing epoch-wise stability. All error values are bad-pixel rates in %.

**Volatility Comparison.** There are large fluctuations in the results across different training epochs. Therefore, we should pay attention not only to peak cross-domain performance but also to result stability. Table 6 indicates that our model exhibits smaller fluctuations than other methods, meaning that it is more robust. The same trend is visualized in Figure 12, where SSPGNet attains both the lowest mean error and an almost-invisible standard-deviation whisker on every benchmark. We attribute these improvements to two main factors: (1) the large model provides robust feature representations, which help the model perform more stably; (2) as shown in Figure 7, the predicted sparse disparity map remains stable in both density and accuracy, which helps ensure effective final results. Meanwhile, compared with the sparse disparity in Figure 6, we find that regardless of whether the setting is in-domain or cross-domain, our model can provide an accurate sparse prompt to guide disparity aggregation, indicating strong generalization ability.

## 5. Discussion

**Figure 13:**
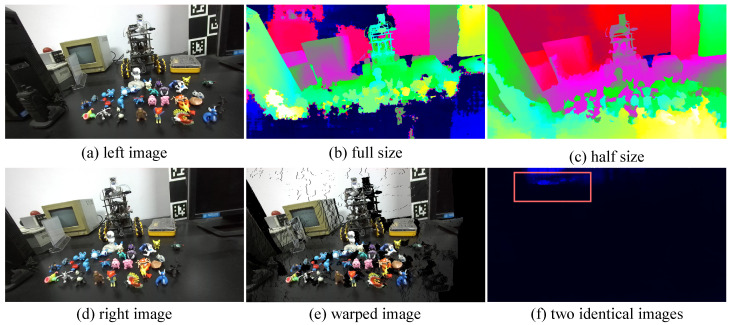
Limitations of the proposed model. It shows that (1) our model cannot handle stereo pairs with a very large disparity range, and (2) when identical images are fed into the model, the output still contains noise.

**Limitations.** Although we have implemented a stereo matching model for open environments, several issues remain unresolved. We have tested the model in the wild and observed strong generalization. However, two core issues still need to be addressed. (1) The disparity range of the current model is limited, for example, to 1∼197. If the target object is captured at close range, the true disparity may exceed 197. As shown in Figure 13b, the model cannot correctly handle regions whose disparity is larger than 197. (2) If we feed two identical images (e.g., two left images) into the network, the expected output should be a zero-disparity map. However, as shown in Figure 13f, the model still produces a disparity map with noticeable noise, suggesting that it may not fully capture the essential concept of stereo matching. Moreover, when we down-sample stereo pairs to fit the predefined disparity range, the model can recover a reasonable disparity map, as shown in Figure 13c. We also warp the left image using the predicted disparity map to reconstruct the right image, and Figure 13e shows that the reconstructed right image is visually reasonable. In related tasks such as multi-view depth estimation, the search range in the cost volume can be adjusted for different depth ranges to obtain appropriate results. In our case, however, simply changing the disparity range still does not fully solve the problem. Overall, despite achieving good generalization, the network still does not seem to fully capture the essential concept of matching.

**Computational Profile.** On a single NVIDIA RTX 3090 at the KITTI input envelope (378×1246), SSPGNet has 313.38 M total parameters (304.37 M frozen in the DINOv2 ViT-L/14 backbone and 9.01 M trainable), requires 2.92 TFLOPs per stereo-pair forward, and runs at 0.609 s per pair. The runtime is therefore competitive with other VFM-based stereo methods, but the model is not real-time on consumer hardware in its current form. For real-time or mobile deployment, replacing the ViT-L/14 backbone with the smaller ViT-S/14 (∼22 M parameters), applying standard ViT compression (quantization, distillation, and structured pruning), or lowering the input resolution would be the most effective starting point; a full mobile-oriented redesign is left to future work.

**Future Work.** The disparity range varies across scenes and focal lengths. Although some methods claim to identify disparity ranges dynamically, they are still limited to specific datasets. Thus, based on the work presented in this paper, we hope to further explore dynamic disparity-range perception and develop a stereo matching model that is more suitable for engineering applications. Meanwhile, we will further study how to help the model better capture abstract concepts such as three-dimensional geometry and stereo correspondence. Beyond stereo matching, the proposed sparse self-prompt mechanism is conceptually generalizable to other dense 3D perception tasks. For multi-view depth estimation, where plane-sweep cost volumes are routinely constructed, multi-view consistency offers an even stronger confidence signal for the sparse prompt, and the proposed cross-shift window attention naturally extends to cross-view interaction across multiple reference frames. For monocular depth estimation, the prompt can be obtained either from external sparse cues (e.g., LiDAR or SfM keypoints, as in depth completion) or from the network’s own internal uncertainty and then propagated to a dense map via VFM-affinity-guided spatial propagation, which is particularly promising for resolving the scale-shift ambiguity of relative depth produced by VFM-based monocular models. We leave a thorough investigation of these extensions to future work.

## 6. Conclusions

In this work, we present SSPGNet, a sparse self-prompt-guided network for stereo matching that targets strong real-world generalization. The method couples (i) a confidence-driven sparse disparity prompt, self-estimated from vision foundation model features, with (ii) a cross-attention-based sparse-to-dense propagation module that progressively refines the prompt into a dense disparity map. Along the way, we also find that tokens from vision foundation models alone do not preserve sufficient pixel-level coordinate information for stereo matching, which motivates the explicit feature transformation step in our pipeline.

Quantitatively, under the cross-domain (zero-shot) protocol, SSPGNet achieves bad-pixel error rates of 3.6% on KITTI 2012 (>3 px), 4.4% on KITTI 2015 (>3 px), 7.6% on Middlebury (>2 px), and 2.1% on ETH3D (>1 px) (Section 4.3), ranking first on three of the four public benchmarks while exhibiting markedly lower volatility across training epochs than prior methods. Moreover, on the in-the-wild stereo pairs collected with a ZED 2 camera, SSPGNet recovers clean disparity maps and faithful 3D reconstructions across diverse indoor and outdoor scenes, demonstrating that the proposed sparse-to-dense prompt mechanism is effective beyond curated benchmarks. We hope that this work, together with the publicly released code and dataset, will encourage further investigation into foundation model-driven stereo perception in real-world deployment scenarios.

## Figures and Tables

**Table 1:** Representative deep stereo matching methods relevant to SSPGNet.The selection spans seminal end-to-end works (2017–2022), domain-generalized stereo matching (2022–2025), and recent vision foundation model and self-supervised stereo works (2024–2026). The last column makes explicit how SSPGNet differs from each prior method.

Method	Year	Key Mechanism	Strengths	Limitations	Gap Addressed by SSPGNet
GC-Net [17]	2017	End-to-end 3D-conv stereo regression	First fully learned stereo network	Pure CNN; no domain generalization design	Replaces CNN backbone with VFM features and adds a sparse self-prompt
CFNet [18]	2021	Cascade pyramid cost volume	Coarse-to-fine accuracy	Sensitive to domain shift; no foundation prior	Sparse prompt acts as a self-supplied coarse cue across domains
CreStereo [10]	2022	Recurrent refinement + group correlation	Strong on real images	Iteration-heavy; not designed for cross-domain	Single sparse-to-dense step replaces dense iterative updates
Mask-CFNet [25]	2023	Masked modeling for stereo	Stable cross-domain accuracy	Capacity bounded by CNN backbone	We move to a vision foundation model backbone
NMRF [20]	2024	Neural Markov Random Field over disparities	Adaptive disparity-search pruning	No foundation model prior	We pair NMRF-style propagation with a VFM-derived sparse prompt
FormerStereo [26]	2024	VFM features + cosine-constrained cost	First VFM-based stereo with strong zero-shot	Evaluated only on curated benchmarks	We add an in-the-wild ZED 2 evaluation and a self-prompt mechanism
DEFOM-Stereo [29]	2025	Mono-depth model + recurrent stereo	Recurrent VFM mono–prior fusion	Iteration-heavy; relies on an external mono-depth model	Self-prompt is internal and feed-forward, no external mono required
ADStereo [23]	2025	Adaptive down-sampling + disparity alignment	Real-time inference with competitive accuracy	Not optimized for cross-domain generalization	Sacrifices marginal speed for substantially stronger zero-shot real-world generalization
DKT++ [31]	2025	Knowledge-transfer fine-tuning with robustness preservation	Preserves cross-domain robustness after target-domain fine-tuning	Requires target real-domain data; not pure synthetic-only protocol	We achieve competitive cross-domain accuracy without any real-domain training
SMFormer [43]	2026	VFM features + augmentation-consistency self-supervision	Strong self-supervised stereo with competitive zero-shot results	Relies on a complex augmentation regimen	Self-prompt confidence replaces external augmentation regularization

**Table 2:** Ablation study of our model. It shows that our model improves performance in both in-domain and cross-domain settings. Runtimes are measured on a single NVIDIA RTX 3090 (24 GB) at an input image resolution of 378×1246 pixels (the smallest multiple-of-14 envelope covering KITTI’s raw 376×1241 resolution, as required by the DINOv2 ViT-L/14 patch size); the output disparity map has the same spatial resolution as the input. Each runtime entry is the average of 10 forward passes after 3 warm-up iterations. All error values are bad-pixel rates in %.

FTM	SPM	SF (>3 px) [%]	KT-12 (>3 px) [%]	Runtime (s)
✘	✘	5.30	5.32	0.54
✔	✘	4.00	4.07	0.55
✘	✔	3.98	4.06	0.68
✔	✔	**3.20**	**3.62**	0.70

✔: component included; ✘: component excluded; **bold**: best score per column.

**Table 3:** Accuracy under different confidence thresholds. It shows that the model predicts accurate sparse disparity maps. All error values are bad-pixel rates in %.

*t*	Cre >3 px	SF >3 px	KT-12 >3 px [%]	KT-15 >3 px [%]	MB >2 px [%]	ET >1 px [%]
0.08	1.9	2.1	4.6	4.0	8.7	2.7
0.10	1.6	1.9	3.7	3.4	8.2	2.5
0.12	1.4	1.6	2.8	2.7	8.1	2.3
0.15	1.2	1.5	1.6	1.8	7.8	1.7

**Table 4:** Comparison of different backbones, including a convolutional neural network (CNN) baseline. It indicates that DepthAnything v2 is more suitable for stereo matching tasks. All error values are bad-pixel rates in %.

Backbone	Size	SF (>3 px) [%]	KT-12 (>3) [%]
CNN	-	3.90	4.98
DINO v2	S	3.65	4.84
DINO v2	L	3.22	4.54
DepthAnything v2	S	3.61	3.79
DepthAnything v2	L	**3.20**	**3.62**

**Table 5:** Peak cross-domain generalization evaluation. It shows that our model achieves state-of-the-art cross-domain performance. All error values are bad-pixel rates in %.

Method	Year	KT-12 (>3 px) [%]	KT-15 (>3 px) [%]	MB (>2 px) [%]	ET (>1 px) [%]
PSMNet [47]	2018	15.1	16.3	26.9	23.8
CFNet [18]	2021	4.7	5.8	15.3	5.8
LacGwcNet [45]	2022	6.0	5.7	18.3	6.3
GF-PSMNet [39]	2022	5.0	5.3	17.6	11.4
Mask-PSMNet [25]	2023	5.3	6.0	15.8	10.6
Mask-LacGwcNet [25]	2023	5.7	5.6	16.9	5.3
HVT-RAFT [48]	2023	3.7	5.2	10.4	3.0
NMRF [20]	2024	4.2	4.9	7.5	3.8
Selective-IGEV [21]	2024	4.5	5.6	9.2	5.7
Former-RAFT-DAM [26]	2024	3.9	5.1	8.1	3.3
DEFOM-Stereo [29]	2025	3.7	4.9	11.9	2.3
CST-IGEV [22]	2025	4.1	5.2	9.8	4.2
ADStereo [23]	2025	7.4	8.7	14.7	6.8
SMFormer [43]	2026	4.1	4.7	8.1	2.9
SSPGNet	2026	3.6	4.4	7.6	2.1

**Table 6:** Volatility evaluation of cross-domain generalization. It shows that our model is more stable across different epochs. All error values are bad-pixel rates in %.

Method	Year	KT-12 (>3 px) [%]	KT-15 (>3 px) [%]	MB (>2 px) [%]	ET (>1 px) [%]
PSMNet [47]	2018	10.4±3.50	14.70±1.03	22.40±7.54	17.60±1.46
GANet [49]	2019	8.90±0.75	12.10±1.19	19.10±9.24	12.10±1.23
DSMNet [50]	2020	5.90±1.14	6.60±0.18	19.70±3.64	7.12±1.65
GF-PSMNet [39]	2022	6.00±0.89	5.70±0.58	17.80±1.87	12.30±1.59
LacGwcNet [45]	2022	9.17±10.46	8.37±9.24	18.28±0.51	7.99±1.37
CFNet [18]	2021	5.82±0.13	6.56±0.19	15.16±0.90	7.24±0.30
Mask-PSMNet [25]	2023	6.66±0.66	6.36±0.31	16.56±0.94	11.4±0.91
Mask-LacGwcNet [25]	2023	6.57±0.30	6.08±0.23	17.30±0.89	6.57±1.03
Mask-CFNet [25]	2023	5.03±0.03	6.08±0.07	12.82±0.37	6.63±0.21
Former-PSMNet [26]	2024	4.30±0.20	5.20±0.13	11.50±0.30	9.40±1.52
SSPGNet	2026	3.65±0.01	4.45±0.03	7.66±0.05	2.29±0.20

**Figure A1:**
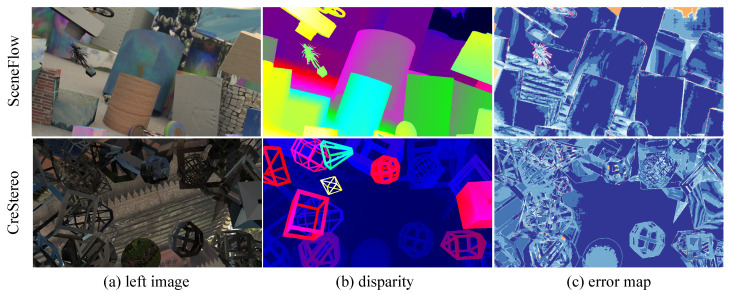
Example results in the source domain. Our model predicts clear disparity maps on the pre-training datasets.

**Figure A2:**
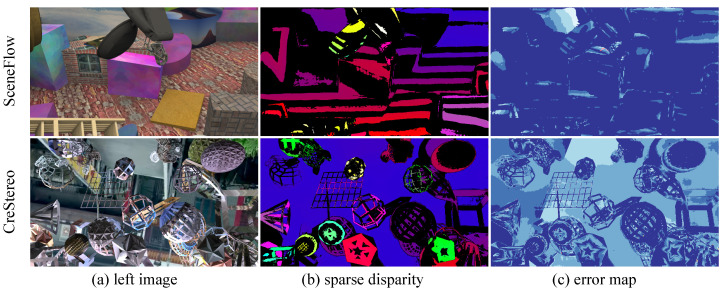
Sparse disparity in the source domain. It shows that the model can generate a high-quality prompt in the source domain.

**Figure A3:**
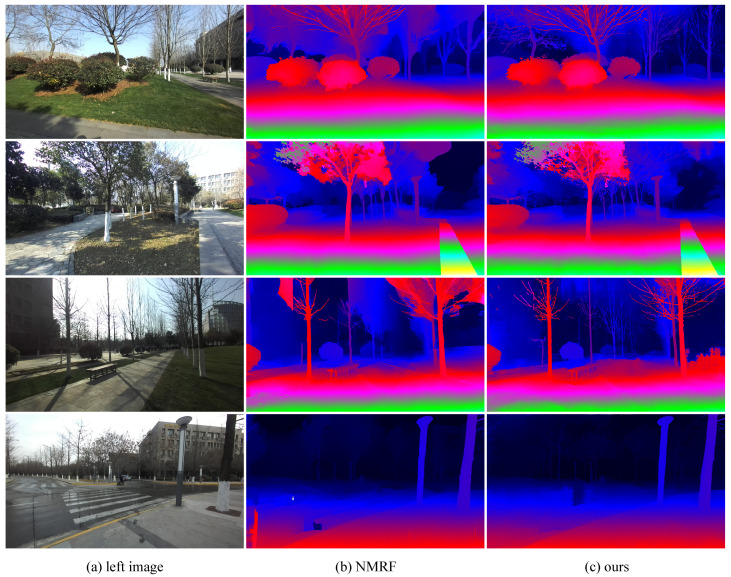
Comparison of results on the ZED dataset.

**Figure A4:**
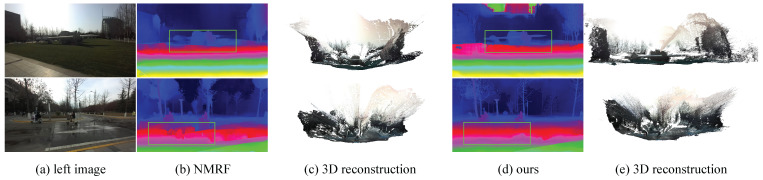
Example results in the wild. We collected stereo pairs in the wild using a ZED 2 camera to test the model’s generalization. The results show that our model can obtain good disparity predictions in the wild, indicating its practical applicability.

**Figure A5:**
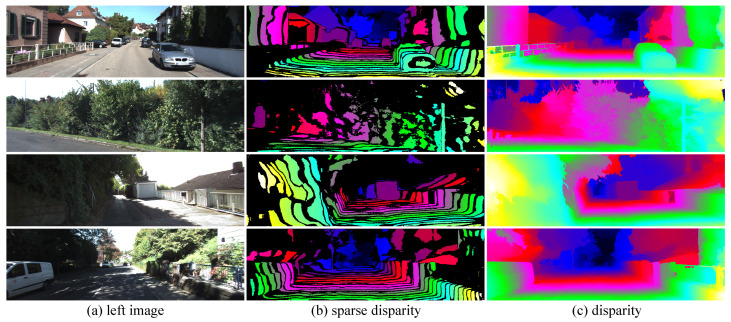
Results on the KITTI 2012 dataset.

**Figure A6:**
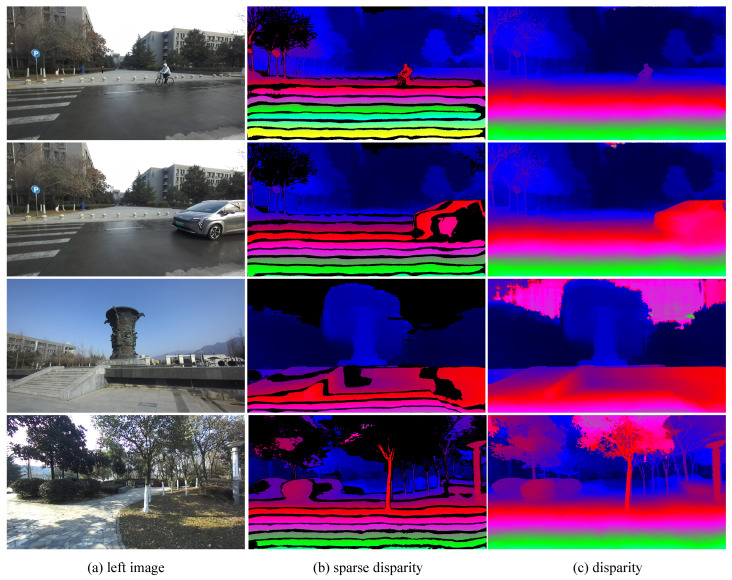
Results on the ZED dataset.

**Figure A7:**
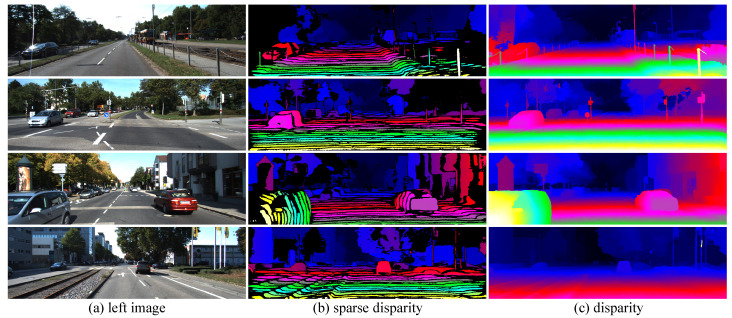
Results on the KITTI 2015 dataset.

**Figure A8:**
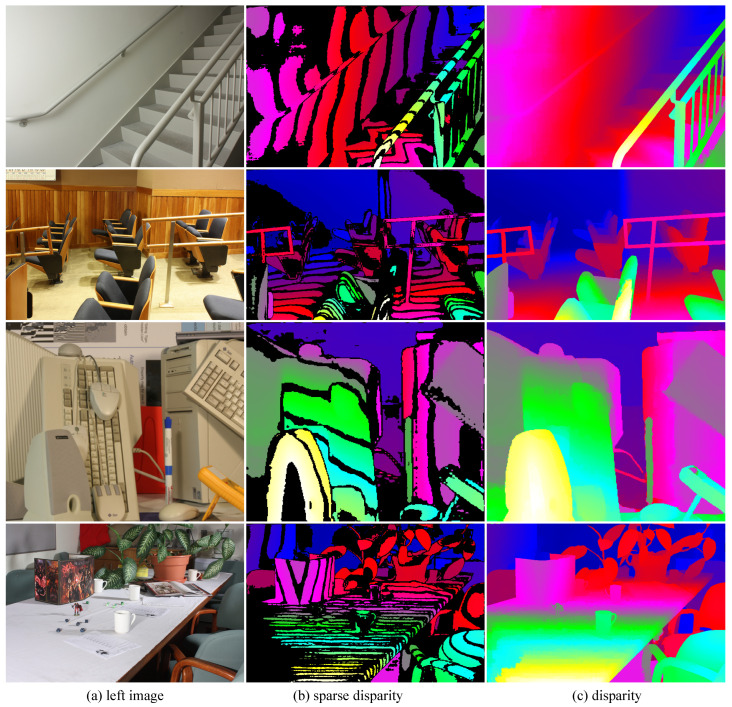
Results on the Middlebury dataset.

**Figure A9:**
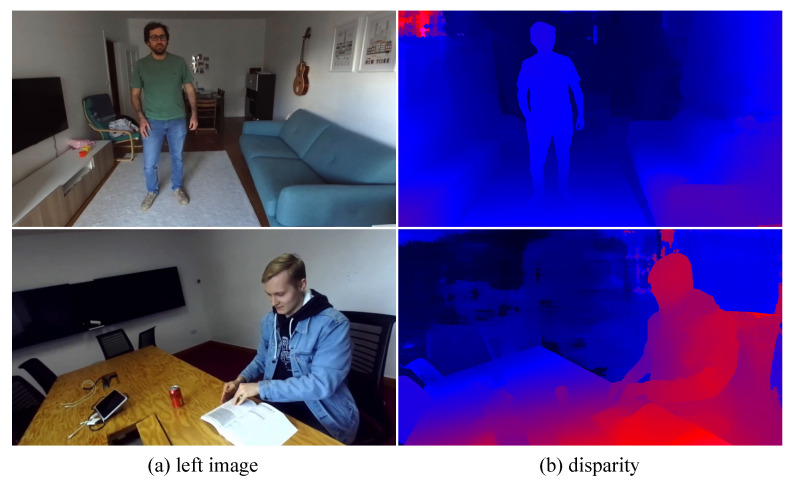
Results on the Dynamic dataset.

**Figure A10:**
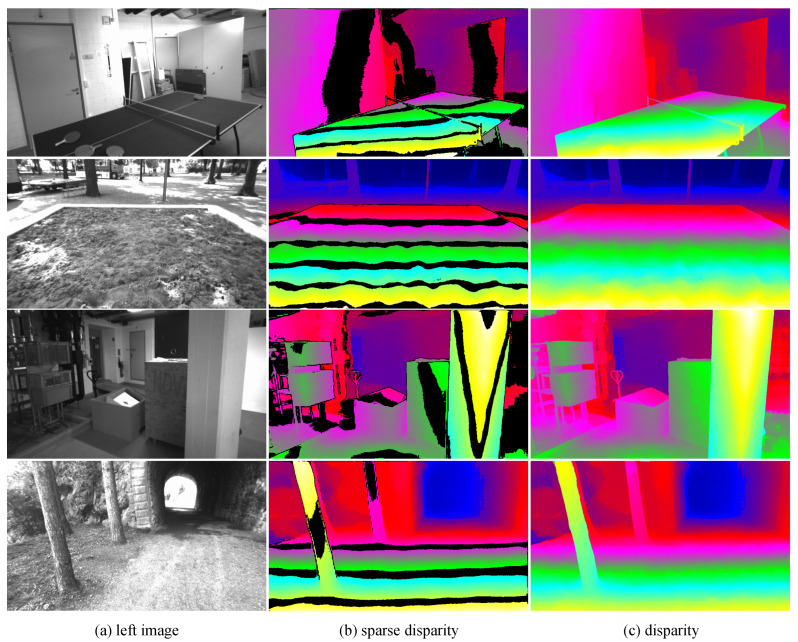
Results on the ETH3D dataset.

**Figure A11:**
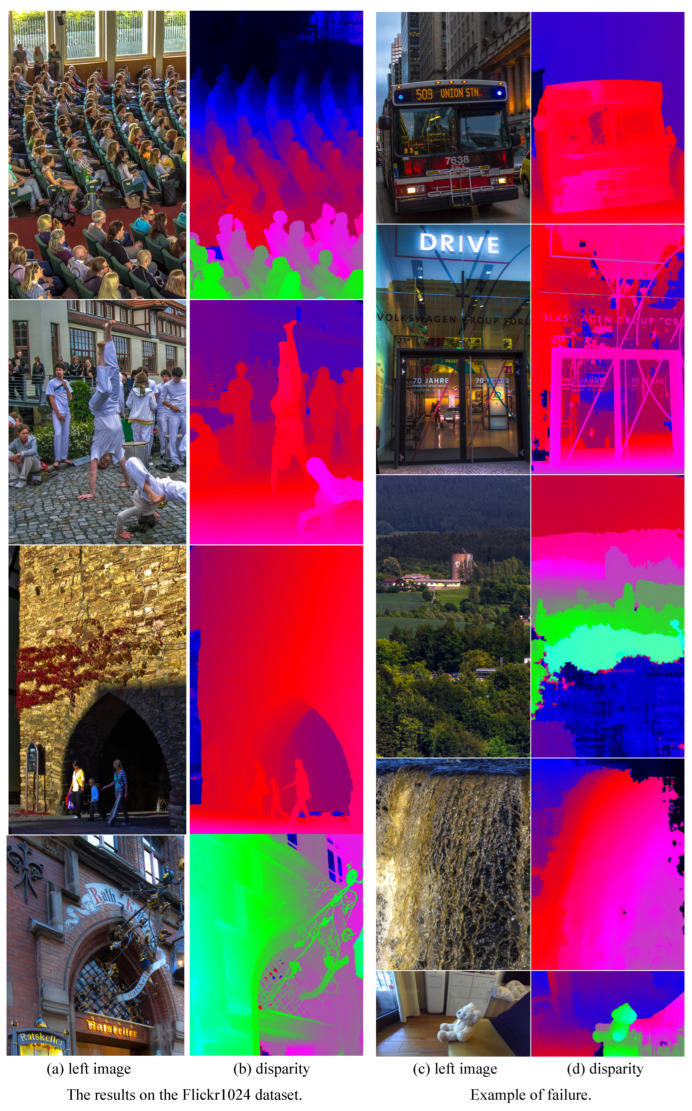
Results and a failure case on the Flickr1024 dataset.

## Data Availability

The source code of the proposed SSPGNet, together with the in-the-wild stereo dataset collected using a ZED 2 camera that supports the findings of this study, are openly available on GitHub at https://github.com/Archaic-Atom/SSPSNet (accessed on 13 May 2026).

## Appendix A

This Appendix A first provides implementation details in Appendix A.1. Then, we present more experiments in Appendix A.2 We show more visualization results in Appendix A.3. Finally, we discuss systematic failure modes and error patterns of SSPGNet in Appendix A.4.

### Appendix A.1. Implementation Details

In this paper, we implement our model using PyTorch. According to our goal, we determine the hyper-parameters and training schedule as follows.

**Hyper-parameters.** We set the maximum disparity to D=196 to ensure that nearly all possible disparity values in the images can be covered. We use the Adam optimizer $\left(\beta_{1}=0.9,\beta_{2}=0.999\right)$ to optimize the model. During training, we set a mini-batch size of three image pairs per GPU (18 on 6 GPUs). In addition, the patch size p=14 is determined by the DINOv2/DepthAnything v2 backbone (ViT-L/14) and cannot be altered without re-training the foundation model; the foundation model layer indices {4, 11, 17, 23} are justified in Section 3.2. The four confidence thresholds t∈{0.08, 0.10, 0.12, 0.15} are reported in the ablation of Table 3; we use t=0.10 during training and the densest reliable setting t=0.15 at test time. The number of Laplacian components *K* in Section 3.2 adopts the default of [46]. The affinity matrix has 8 channels because they correspond to the eight neighbors of a 3×3 spatial propagation kernel (one per non-center cell), as dictated by the underlying CSPN-style propagation.

**Training schedule.** We train models only on the SceneFlow or CreStereo dataset. The learning rate is initially set to $1\times 10^{-3}$ for 50 epochs and then reduced to $1\times 10^{-4}$ for the remaining 10 epochs. For data augmentation, we use random cropping, which randomly crops left–right images and the corresponding disparity map to 518×266.

### Appendix A.2. Additional Experiments

**Different Pre-training Datasets.** First, we use our model with the DepthAnything v2 feature extraction module as the baseline. Then, we train our model on two large synthetic datasets (CreStereo and SceneFlow) to compare peak cross-domain performance, as listed in Table A1. Two conclusions can be drawn: (1) the pre-training dataset affects cross-domain performance because of differences in disparity ranges and image styles; (2) among the two datasets, Cre-Stereo helps our model achieve better cross-domain performance, especially for in-the-wild generalization. Therefore, we use the model pre-trained on Cre-Stereo for comparison with state-of-the-art methods.

**Table A1:** Comparison of different pre-trained datasets. Different datasets affect cross-domain performance because of differences in disparity ranges and image styles.

Pre-Trained Dataset	KT-12 (>3 px) [%]	KT-15 (>3 px) [%]	MB (>2 px) [%]	ET (>1 px) [%]
SceneFlow [11]	4.6	4.7	7.9	2.5
Cre-Stereo [10]	3.6	4.2	7.6	2.1

**More Results in the Source Domain.** We present the final disparity and sparse disparity results in Figure A1 and Figure A2. Our model predicts clear disparity maps and high-quality prompts in the source domain. It is worth noting that even in the source domain, our sparse prompt performs well in challenging areas such as occluded or repetitive-texture regions.

**More Results in the Wild.** We show the predicted disparity maps and the reconstructed point cloud in the wild. As shown in Figure A3 and Figure A4, our method preserves scene details more effectively. For example, the sword in the sculpture is recovered more completely in our disparity map, while it is partially missing in the results of other methods. Likewise, the disparity of the ground, which contains repetitive textures, is also better preserved by our method. These results further demonstrate the strong generalization ability of our model and its compatibility with ZED 2 cameras.

### Appendix A.3. More Visualization Results

In this section, we provide qualitative results on in-the-wild scenes and public datasets, including KITTI 2012 and 2015, ETH3D, Middlebury, ZED, Dynamic, and Flickr1024, as shown from Figure A5 to the left side of Figure A11. Our model outputs sparse disparity maps with clear boundaries and high-quality dense disparity maps. We also present failure cases on the right side of Figure A11. These experimental results indicate that our method has strong generalization ability and the potential to be directly applied to binocular systems.

### Appendix A.4. Failure Cases and Error Discussion

Although SSPGNet performs competitively on all the evaluated public benchmarks and on the in-the-wild ZED 2 set, two systematic failure modes remain. Both are illustrated in Figure A11 and discussed below.

**(i) Transparent and reflective surfaces.** The cost-volume formulation implicitly assumes that corresponding pixels in the left and right views share the same color and intensity. Glass and mirror-like surfaces violate this assumption: refraction maps a different scene point through the surface, and specular reflections add a view-dependent appearance layer. SSPGNet has no explicit transparency or material reasoning and therefore produces unreliable disparity on the bus windshield in row 1 of Figure A11 and on the glass storefront/glass-door facade in row 2. A transparency-aware semantic prior, e.g., a learned glass/mirror segmentation that gates the cost-volume contribution in detected transparent regions, is a natural direction for addressing this failure mode.

**(ii) Large regions of repetitive texture.** Stereo matching is fundamentally ambiguous when local appearance is nearly identical at many candidate disparities, because the matching cost then has multiple comparable minima. The confidence-thresholded sparse self-prompt of SSPGNet is, by design, empty in such regions: the cost-volume probability distribution is too flat for any disparity to pass the confidence threshold *t*, so the spatial-propagation step has very few or no anchors to start from and tends to propagate noisy values inward. This pattern is visible in the dense forest foliage of row 3 of Figure A11 and in the waterfall cascades of row 4. A larger-receptive-field contextual aggregation and a multi-scale prompt that searches at coarser spatial scales for disambiguation are two natural directions for future work.
